# Perforation of the Intestine in Typhoid Fever

**Published:** 1903-06-13

**Authors:** 


### PERFORATION OF THE INTESTINE IN TYPHOID FEVER.

The percentage death-rate of all cases of typhoid fever still ranges between 7 and 14 per cent., with an average of 10 per cent. for all cases. In the United States of America the total number of cases of typhoid fever amounts to 500,000 per annum, and the death rate being 10 per cent. of all cases, the total mortality must be nearly 50,000 per annum. Now of these cases of death from typhoid fever it has been shown that fully one-third are due to perforation of the intestine, or a total of over 16,000 cases of fatal perforation. There is but little doubt, also, that of these quite one-half can be saved by surgical interference. (*Cf.* Taylor, *New York Medical Journal*, Feb. 1902.) Medical diagnosis is nowadays more prompt, and surgical progress has advanced to a point where interference in a case of perforation is both useful and necessary. Recovery without operation is a matter of grave doubt, except in a few rare cases of localised infection. In these, where the lesion is confined to the neighbourhood of the cæcum, the case becomes one of perityphlitis. Indeed, recovery in cases with symptoms of intestinal perforation is no satisfactory evidence of recovery from perforation. Since the usefulness of the operation has been established it has been proved, in the course of a series of such cases, that the gravest symptoms of perforation may be present without the actual lesion. On the other hand a rather premature assumption of perforation from vague symptoms has been justified by the discovery of perforation at the subsequent operation.

Nearly 20 years ago the first operation for perforation was undertaken, and up to January 1st, 1900, not more than 200 cases had been published. The operation has been frequently performed since, though it is feared that only reports of successful cases have been recorded. In the diagnosis of intestinal perforation the question of general peritonitis ought not to come into consideration. Many of the so-called symptoms of perforation really belong to general peritonitis, and are indications of that condition alone. It is only by making a clear distinction between perforation and the too frequently associated general peritonitis that a diagnosis of perforation can be accurately arrived at. It is only by such refinements of diagnosis that the disappointment of operating for perforation and finding general peritonitis can be safely avoided.

Again, it has been assumed that the mortality following the operation for perforation has been always due to general peritonitis. This is only in part correct, for many cases die from causes independent of the operation, and at present almost beyond control. Surgical diagnosis in intestinal perforation is now demanded from the point of the utility of surgical interference. But it is the lack of familiarity with the physical condition of the abdomen in typhoid fever that often causes the surgeon to be incapable of giving a well-balanced opinion on a case of suspected perforation. Hence the importance of surgical familiarity with the abdominal conditions in all stages of typhoid fever.

The time at which perforation occurs is, as far as diagnosis goes, of no consequence. It usually happens during the third week, though it may occur as early as the sixth day. But the time of perforation is of great importance with reference to the general condition of the patient, and his chances of withstanding the shock of operation. On the other hand, the severity of the fever may often be overlooked in the prognosis of perforation. Osler states that intestinal perforation is more common when the illness is severe, but from reported cases it can be shown that at least one-half of the instances of perforation occur in mild or even ambulatory typhoid fever. Perforation certainly does occur in the mild cases and must, therefore, be borne in mind. As regards age and sex, it occurs more frequently in young adults, though cases have been recorded among children. (*St. Bartholomew's Hospital Journal*, April, 1903.) Perforation is apparently more common among males than females.

Intestinal hæmorrhage occurring at the same time is apt to mask perforation and render the diagnosis more difficult. A comparatively small number of perforations have been preceded by hæmorrhage from the bowel, and a small percentage of cases of hæmorrhage ultimately go on to perforation of the intestine. So hæmorrhage can be overlooked as an antecedent of perforation.

*Symptoms and Signs of Intestinal Perforation.*—The value of symptoms must depend upon the mental condition of the patient, on his power of appreciation. In the delirious or comatose cases the consideration of symptoms may be entirely set aside. Consequently, in the severe cases, physical signs and phenomena can alone be relied upon.

As far as the symptoms of perforation are of value, pain, sensations of systemic shock, and altered respiration are usually present. Abdominal pain is by far the most important of these symptoms. When present it demands the most careful consideration, and especially so when it appears as a sudden, sharp, and often agonising sensation located in the lower part of the abdomen. The pain may come on gradually and be general at the start, and afterwards become more definitely located. But a localised sensation, gradually becoming general, indicates the progression of the infection from the point of perforation. The location of the pain, usually in the lower part of the abdomen, and its persistence, are only points of secondary importance.

Sensitiveness, closely allied to pain, usually accompanies it in cases of abdominal pain. But the sensitiveness may be more localised than the pain, and hence assist in more definitely locating the lesion. This degree of accentuation is of some use as a symptom of perforation. The symptoms of shock are quite general, and only of importance when taken with the other signs and symptoms of the case.

*Signs.*—In considering the signs of perforation, it must again be emphasised that they are not those of general peritonitis, and the diagnosis of general peritonitis is not the diagnosis of perforation. The signs of perforation include (1) muscular resistance and presence of gas outside the intestine; (2) general systemic indications, *e.g.* vomiting, pulse, temperature, and blood conditions.

Muscular resistance is important in all cases of acute intra-abdominal lesions. Pain and sensitiveness are usually associated with muscular resistance, but the muscular resistance is present in patients either comatose or delirious. The muscular resistance is usually general, though often accentuated in the lower half of the abdomen. Such muscular resistance is rarely absent in perforation of the intestine. Even when present beforehand, it is probably always increased.

Distension and tympany are of little import *per se.* They are only confirmatory evidence of spreading peritoneal infection. Abdominal gas *outside* the intestine is, however, when demonstrated with certainty, practically a proof of perforation. Obliterated liver dulness is not a sign of free gas in the peritoneum. It has been proved again and again to be due to a distended colon, or over-filled small intestine, quite apart from any free gas in the peritoneum. Hence alterations of liver dulness can only arouse suspicion of perforation.

Nausea and vomiting only occur infrequently, in less than 25 per cent. of the cases. Their presence is of slight confirmatory value. Altered respiratory movements are of considerable import. When perforation has occurred respiration is invariably distinctly thoracic in character. It is recognisable, even when the mental condition of the patient prevents any attention being paid to symptoms.

Alterations in body temperature usually accompany perforation. Rigors are an infrequent accompaniment. Many cases show a fall of 2° Fahr., really of little value, in the irregular temperature curve of typhoid fever. A decided drop of temperature is more frequently met with in hæmorrhage from the bowel than in perforation of the intestine.

The pulse gives indications of the greatest importance. (1) There is a sudden rise in the pulse-rate of 20 beats or more per minute. (2) The quality of the pulse is also altered. The tension is reduced, and the artery is more easily compressible. These changes are more noticeable in the less severe cases of typhoid fever, but are always more sudden in perforation than in hæmorrhage from the bowel.

*Blood Changes.*—It is doubtful if the leucocyte count is affected by perforation itself, though a leucocytosis may occur in one to six hours afterwards. But unfortunately a sudden and extensive leucocytosis may take place without any perforation of the intestine. Still some consider a "wave of leucocytosis" to follow perforation. The signs from the blood-count are thus of little moment. Systemic shock is always present, though perhaps often unrecognised.

But the pallid countenance, drawn facial expression, slight cyanosis, and sudden perspiration are all evidence that goes to make a striking picture of perforation of the intestine.

